# A Simple Insightful Approach to Investigating a Hospital Standardised Mortality Ratio: An Illustrative Case-Study

**DOI:** 10.1371/journal.pone.0057845

**Published:** 2013-03-05

**Authors:** Mohammed A. Mohammed, Andrew J. Stevens

**Affiliations:** 1 Primary Care Clinical Sciences, University of Birmingham, Birmingham, United Kingdom; 2 Public Health, Epidemiology & Biostatistics, University of Birmingham, Birmingham, United Kingdom; University Medical Center Rotterdam, The Netherlands

## Abstract

**Background:**

Despite methodological concerns Hospital Standardised Mortality Ratios (HSMRs) are promoted as measures of performance. Hospitals that experience an increase in their HSMR are presented with a serious challenge but with little guidance on how to investigate this complex phenomenon. We illustrate a simple penetrating approach.

**Methods:**

Retrospective analysis of routinely collected hospital admissions data comparing observed and expected deaths predicted by the Dr Foster Unit case mix adjustment method over three years (n = 74,860 admissions) in Shropshire and Telford NHS Trust Hospital (SaTH) constituting PRH (Princess Royal Hospital) and RSH (Royal Shrewsbury Hospital); whose HSMR increased from 99 in the year 2008/09 to 118 in the year 2009/10.

**Results:**

The step up in HSMR was primarily located in PRH (109 to 130 vs. 105 to 118 RSH). Disentangling the HSMR by plotting run charts of observed and expected deaths showed that observed deaths were stable in RSH and PRH but expected deaths, especially at PRH, had fallen. The fall in expected deaths has two possible explanations–genuinely lower risk admissions or that the case-mix adjustment model is underestimating the risk of admissions perhaps because of inadequate clinical coding. There was no evidence that the case-mix profile of admissions had changed but there was considerable evidence that clinical coding process at PRH was producing a lower depth of coding resulting in lower expected mortality.

**Conclusion:**

Knowing whether the change (increase/decrease) in HSMR is driven by the numerator or the denominator is a crucial pivotal first step in understanding a given HSMR and so such information should be an integral part of the HSMR reporting methodology.

## Introduction

Comparison of hospital mortality statistics, usually in the form of a hospital standardised mortality ratio (HSMR) are used in several countries [1]–[5] under the controversial premise that variations in HSMR reflect differences in quality of care [6]–[7], [9]. HSMRs were introduced by Jarman et al [1] for National Health Service (NHS) hospitals in England in 1999 and further developed by the Dr Foster Unit [8] and subsequently publically reported annually [9] by Dr Foster Intelligence [10].

The HSMR is a ratio of observed and expected deaths, and is usually multiplied by 100 for convenience. Expected deaths are determined from statistical models which adjust for a variety of patient risk-factors obtained from routinely collected hospital administrative databases. Although serious methodological concerns remain over case-mix adjustment and HSMRs [11]–[14] hospitals labelled with a high or escalating HSMR are presented with a grave challenge, but often with little practical guidance on how to investigate this complex phenomenon. This paper illustrates a practical method that can be used to aid understanding of what does/does not lie behind a given HSMR. Our method relies on visualisation of data [15] using simple run charts [16] and crucially, involves separate analyses of the numerator (observed deaths) and denominator (expected deaths) that makes up the HSMR, as suggested by Parry et al [16].

Our case-study involves Shropshire and Telford NHS Trust Hospitals (SaTH). In 2008/09 the Dr Foster HSMR for SaTH was 99, but in 2009/10 this jumped to 118 (19% increase) and now labelling SaTH as a high mortality hospital, because the observed deaths were now significantly higher than expected after case-mix adjustment. As SaTH is made up of two hospitals - the Royal Shrewsbury Hospital (RSH) and the Princess Royal Telford Hospital (PRH) - we took the opportunity to undertake comparative analyses. Although our case-study focuses on an increasing HSMR, the methods we illustrate should be used to improve our understanding of decreasing or stable HSMRs.

## Methods

Since the HSMR is the ratio of observed/expected deaths, an increase in the HSMR can come from an increase in the numerator (observed deaths) or a decrease in the denominator (expected deaths). So, our investigation was guided by the following generic reasoning. If the increase in HSMR is primarily driven by an increase in observed deaths, then there are two possible candidate explanations, which are not mutually exclusive. (1) A deterioration in quality of care and/or (2) an increase in patient severity not reflected in the expected mortality. If the increase in HSMR is primarily driven by a fall in expected deaths, then again there are two possible explanations which are also not mutually exclusive. (1) A decrease in patient severity accompanied by a deterioration in quality of care and/or (2) that the case-mix adjustment model is underestimating the risk of admissions (eg perhaps because of inadequate clinical coding – an acknowledged phenomenon [8], [17]).

We used SaTH hospital admissions data (n = 74,860) from the Dr Foster Real Time Monitoring computer system for the three year period from April 2007 to March 2010 (36 months). These data include patient age, gender, deprivation quintile, primary diagnosis, and the Charlson Index of co-morbidities [17] which is derived from clinically coded secondary diagnoses. The methodology to produce the Dr Foster HSMR is described elsewhere [9], [18]. Using these data we additionally derived a measure of completeness of the clinical coding process, by calculating the number of International Classification of Diseases (ICD)-10 codes (excluding the primary diagnosis) per admission, which we refer to as the coding depth. For each year we obtained the observed and expected numbers of death on a monthly (n = 36) basis. There are several candidate case-mix indicators available – eg age, gender, deprivation and the Charlson Index of co-morbidities. We focus on %died, %emergency admissions, mean age (years), the mean Charlson index and the mean length of stay (days) as our primary indicators of patient case-mix profiles.

We plotted observed and expected deaths as mean centered (to aid visualisation) run charts over the 36 months where we regarded a run of seven consecutive points above/below zero as unusual [13]. We used the insights from these run charts to develop and investigate hypotheses that might credibly explain the step change in the HSMR at SaTH. This preliminary investigation is restricted to data available at the desktop (ie within the Dr Foster HSMR data frame) and involves separate monthly run charts for the two hospitals (RSH & PRH) that make up SaTH.

This study was undertaken as part of SaTH’s regular audit and evaluation work using routinely collected administrative hospital data. All data was de-identified before analysis.

## Results

**Figure 1:**
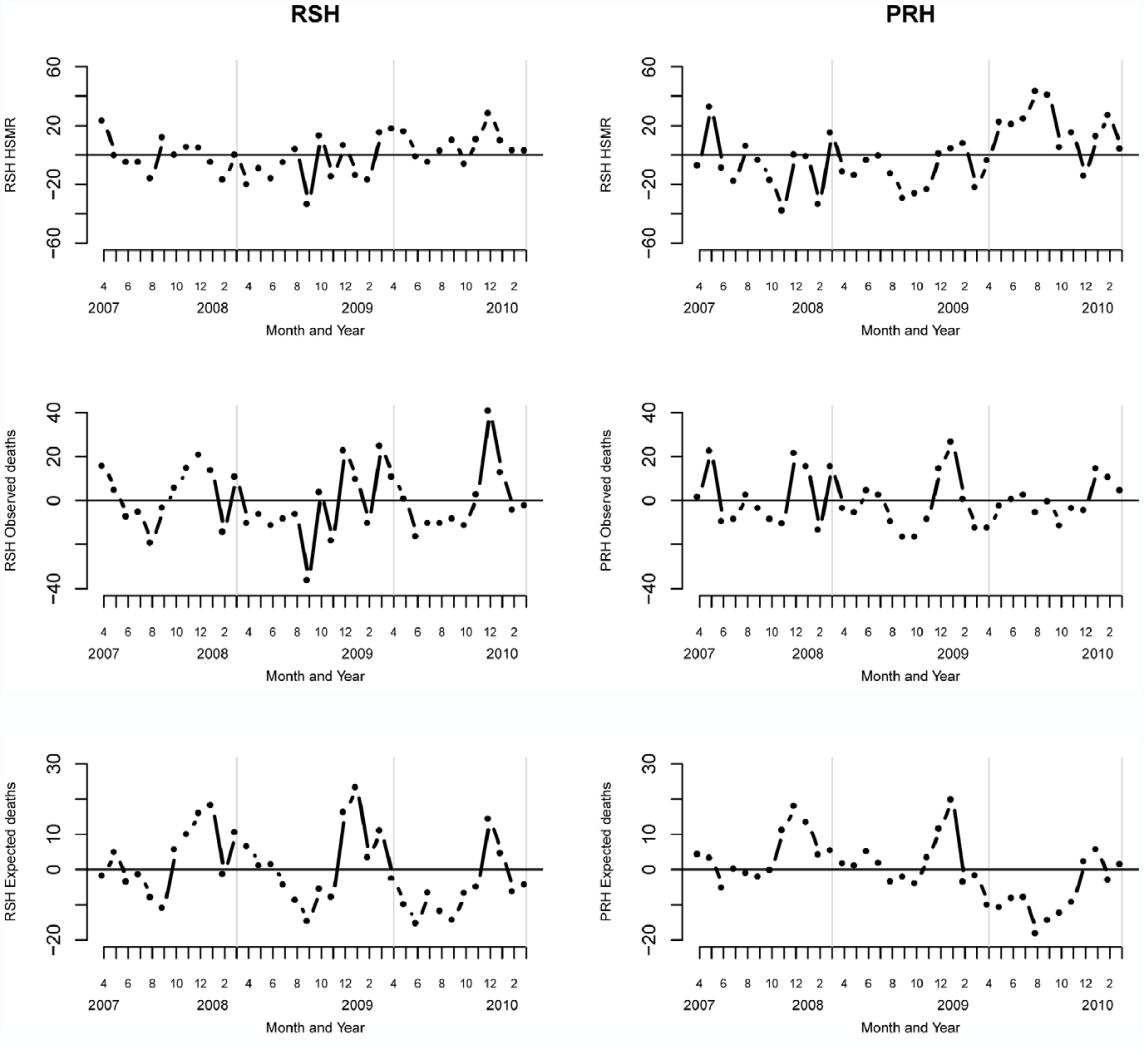
Monthly HSMRs for RSH and PRH over three years. In all panels vertical grey lines partition the three years (April 2007– March 2008; April 2008– March 2009; April 2009– March 2010).

Table 1 shows the HSMR for SaTH and its two hospitals over a three year period. It appears that the majority of the increase in HSMR is located in PRH in year 3 when its HSMR jumped from 105 to 130, and this was confirmed further by plotting the HSMRs over time for SaTH and its two hospitals (see top panel Figure 1). It is worth noting that if the HSMR was to be reported separately, then it would be PRH that would be an “outlier” (according to the Dr Foster Real Time Monitoring system, version 8.0, funnel plot as a guide) and not RSH, and so we focused our attention on understanding the step change in PRH (Table 1).

**Table 1:** HSMRs for SaTH and its two constituent hospitals over three years.

	HSMR	HSMR	HSMR
Hospital	Year 1: Apr 2007– Mar 2008	Year 2: Apr 2008– Mar 2009	Year 3: Apr 2009– Mar 2010
SaTH	105	99	118
RSH	101	95	110
PRH	109	105	130

We then examined observed and expected deaths separately for RSH and PRH (Figure 1 middle and lower panel). We found that observed deaths were fairly stable in both hospitals, but expected deaths showed some unusual patterns in year 3. We see a remarkable run of 8 consecutive points below the mean (Apr 2009 month 25 to Nov 2009 month 32) in RSH and in PRH we see a remarkable run of 10 consecutive points below the mean (Feb 2009 month 23 to Dec 2009 month 33), which no equivalent patterns in observed deaths. In other words, the dominant signal for the increase in HSMR in year three at SaTH can be located to an extraordinary drop in expected numbers of deaths with no material change to the underlying number of observed deaths in the same period.

This phenomenon (of relatively stable observed deaths and falling expected deaths) has at least two, not mutually exclusive, candidate explanations - 1) patients admitted during this period are not as sick as in the previous two years but have nevertheless experienced higher than expected mortality (perhaps because of poorer quality of care), and/or 2) the true risk of dying has not been sufficiently well captured in the HSMR calculation and so the drop in expected mortality is an artefact resulting perhaps from less complete clinical coding. We undertook further investigations using run charts to further explore, within the limits of a desktop exercise, the credibility of these hypotheses.

### The Less Severe Admissions Hypothesis

**Figure 2:**
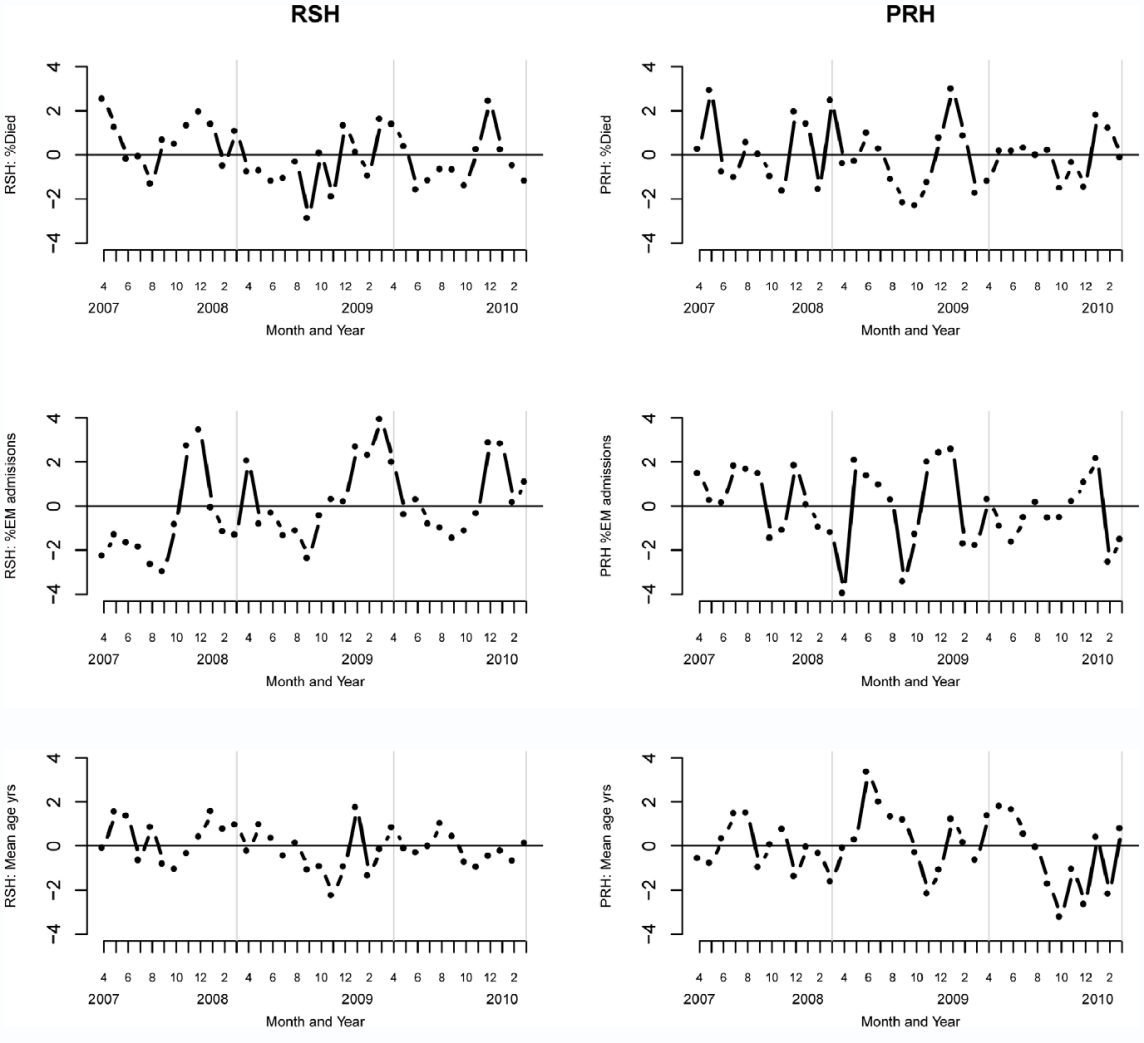
Case-mix profile of admissions to RSH (left panel) and PRH (right panel). First row (top):%Died. Second row: %Emergency admissions. Third row: Mean age (years). In all panels the vertical grey lines partition the three years.

We relied on %died, %emergency admissions, mean age (years), the mean Charlson index and the mean length of stay (days) as our primary indicators of patient case-mix profiles. Figure 2 (top row) shows that %died has remained largely stable, that %emergency admissions (middle row) are also relatively stable, at least over years 2 and 3, and that this is associated with no major changes in the underlying mean age (years) of the admissions (bottom row).

**Figure 3:**
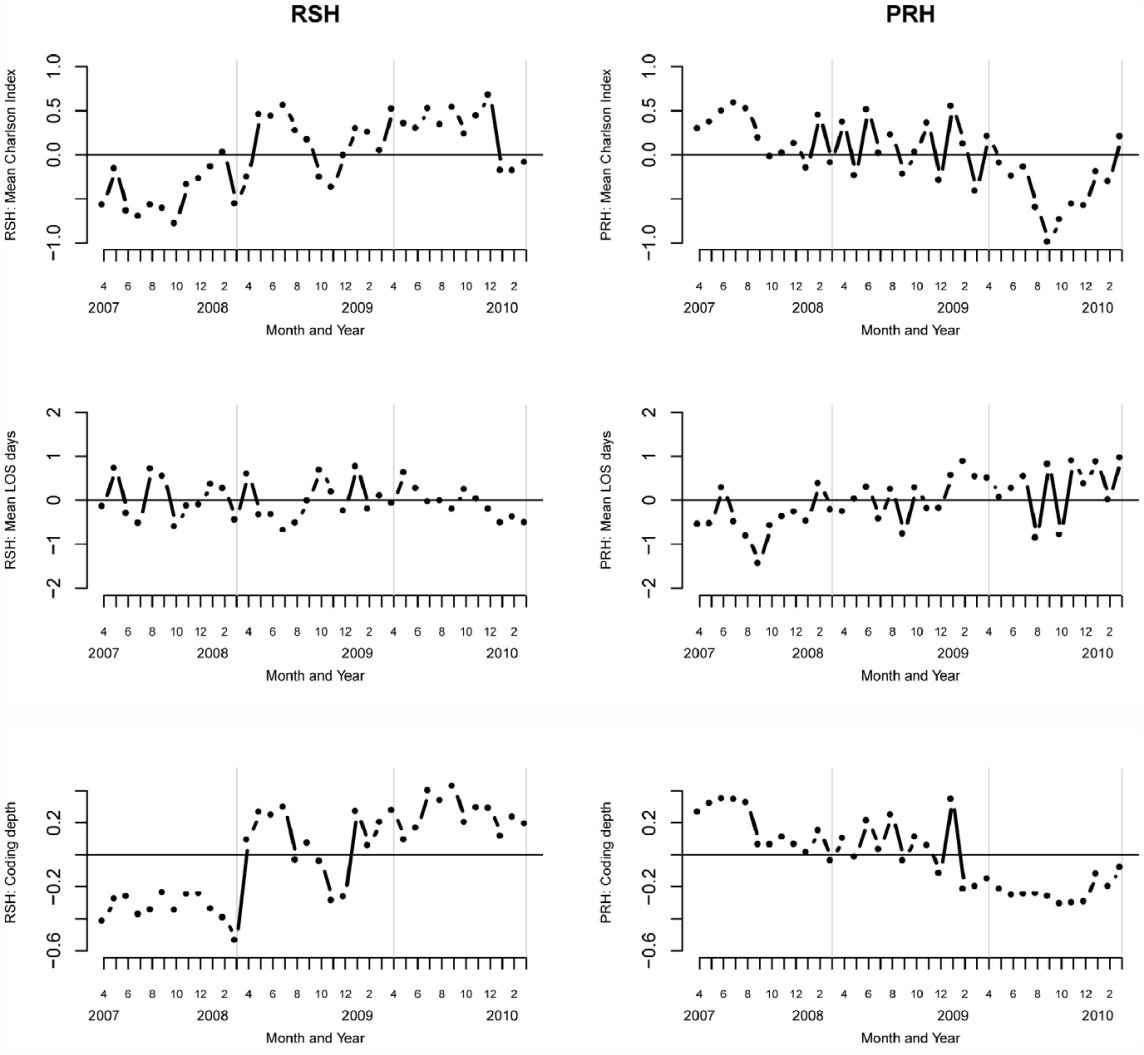
Additional case-mix profile of admissions to RSH and PRH. Top row: Mean Charlson Index. Middle row: Mean length of stay (days). Bottom row: Mean coding depth. In all panels the vertical grey lines partition the three years.

Further case-mix profiles are seen in Figure 3. There is clear evidence that the mean Charlson index (Figure 3, top row) in RSH has seen a step increase and that at PRH it has been steadily declining with a clear dip in year 3. If this pattern in the Charlson index is an accurate reflection of a genuine change in patient case-mix profiles, then we would (all else being equal), usually expect the length of stay to be longer for sicker patients and conversely shorter for the less sick. However the length of stay profiles (Figure 3, middle row) at RSH and PRH do not fit with this view. At RSH length of stay has been relatively stable and at PRH it has been steadily rising.

The plots considered so far then are not consistent with the view that patients admitted in year 3 are “less sick” at RSH or PRH in year 3 than in the previous two years and therefore call into question the validity of the expected numbers of death in year 3 as reliable measures of patient case-mix.

### The HSMR Model Underestimates the Risk of Admissions Hypothesis

We now explore the hypothesis that the HSMR model underestimates the risk of admissions because of changes in the clinical coding process. Figure 3 (bottom row) shows the mean depth of clinical coding for RSH and PRH. Remarkably (given the above), the step up/down in the mean Charlson score at RSH and PRH is accompanied by a near simultaneous step up in depth of coding at RSH and a corresponding trend down at PRH. The operational reasons for RSH and PRH diverging in their depth of coding in year 3 need further investigation. Nevertheless, taken as a whole, there is evidence that the fall in expected number of deaths at PRH is an artefact of the reduced depth in clinical coding and it is likely that the expected number of deaths at PRH in year 3 are an underestimate because the depth of coding has decreased and this has led to lower Charlson comorbidity scores.

## Discussion

Given the profound implications for hospitals with high, medium or low HSMRs, it is important that we do not prematurely over interpret the HSMR, but instead adopt a scientific process to investigate a given HSMR. However understanding variability in hospital mortality and separating signals from noise is a complex task and hospitals appear to have little guidance on how to better understand increasing, decreasing or stable HSMRs. Our case study suggests three simple, but useful tactics for understanding HSMRs, which share much with advice given by Parry et al [18] on outliers. (1) Separately consider the numerator (observed deaths) and denominator (expected deaths) of the HSMR, because this shapes the subsequent investigation process. (2) Visualise the data using simple run charts, because visualisation is critical to data analysis - “It provides a front line of attack, revealing intricate structure in data that cannot be absorbed in any other way. We discover unimagined effects, and we challenge imagined ones.” [12]. (3) Make comparisons with other hospitals over the same time period, enabling a form of controlled comparison.

When applied to our case-study we learned that the 2009/10 increase in the HSMR at SaTH was located primarily at PRH (and not RSH), was driven by falls in the expected mortality and not a rise in observed mortality and appears to be credibly explained by changes to clinical coding depth as opposed to genuine changes in lower risk admissions. This calls into question the reliability of the expected numbers of death in PRH in 2009/10, especially as there has been no discernable change in the case-mix profiles in either RSH or PRH in the same period. So there is credible evidence that some portion (which cannot easily be quantified) of the step up in HSMR at PRH in 2009/10 was due to a reduction in clinical coding depth and this finding is consistent with known limitations of the HSMR [8], [17], [19].

It is not the first time that a systematic investigation of a high case-mix adjusted mortality has identified a benign credible explanation. An analysis of case-mix adjusted mortality following paediatric cardiac surgery [20] identified Oxford Radcliffe Hospitals NHS Trust as having higher than expected mortality over three epochs but the explanation turned out to relate to incomplete case ascertainment in the HES data base leading to the ironic conclusion that Oxfords “…good data from hospital episode statistics may have paradoxically contributed to its outlier status.” [21]. Another example was the analysis of case-mix adjusted morality following the crimes of Dr Harold Shipman [22] which identified eleven GPs as having unusually high death rates, more or less comparable to those of Shipman [23], but subsequent investigations found that the excess mortality was adequately explained by taking account of the proportions of patients dying in nursing homes [24], [25]. Indeed, evidence from a systematic review [26] suggests that case-mix adjusted mortality rates are an unreliable indicator of quality of care and Lilford et al contend that to reason otherwise is fallacious [27].

Nevertheless, understanding variability in observed and expected mortality and separating signals from noise is a complex task and our desktop investigation has limitations. We used run charts as an exploratory graphical device because of their simplicity, although more sophisticated analyses using statistical process control charts [28], [29], time series analyses [15] and statistical modelling [8] may be undertaken. Although we confined our analyses to variables available in the database used by Dr Foster, we did not consider a range of health system related hypotheses and explanations in our illustrative case-study, although in practice these would be pertinent (eg diversion of emergency patients from PRH to RSH). Indeed the differences in clinical coding patterns seen in RSH and PRH also merit further study [20]. We also note that even though RSH is not an outlier in the Dr Foster analyses, the step up in HSMR for RSH from 95 to 110 (16% increase) is interesting and may also merit closer examination. This step up coincides with a remarkable run of below average expected deaths (eight consecutive points below the mean) and relatively stable observed deaths (bar a peak in December) which merits further study. This combination once again raises the spectre of clinical coding but for different reasons. Even though coding depth and the Charlson index at RSH have risen (suggesting sicker admissions), length of stay, observed deaths, mean age and have not risen, suggesting that the deeper coding at RSH is a change in the clinical coding process and not necessarily a genuine reflection of case-mix. SaTH is known to have low levels of co-morbidity coding and palliative care coding which will put upward pressure on their HSMR [17], [20]. RSH also has an exceptionally high mortality month (month 33, Dec 2009) which apparently coincided with the swine flu epidemic, which may also merit further investigation, especially as a similar peak is not seen at PRH. Finally we have demonstrated our approach using the Dr Foster HSMR, but it is likely to be useful with other case-mix adjustment methods [12] as well.

The high stakes associated with high mortality prompted Keogh to question how the issue of identifying potential outliers is handled. He asked “Is a peer reviewed journal the right place for publication? What are the relative responsibilities of authors, reviewers, and editors–particularly when a review process may take several months and delay remedial action?” [20] To this we would add, what are the responsibilities of producers of case-mix adjusted mortality rates?

An interesting response to these issues has been demonstrated by the Queensland model [30] which has incorporated The Pyramid Model of Investigation into its case-mix adjusted mortality monitoring programme. This has the advantage of making explicit the investigation steps that must be undertaken when faced with a high mortality and involves an incremental approach starting at the base of the Pyramid (with questions about the data) and then moving higher up the Pyramid (towards quality of care) where this is justifiable. In a similar manner, this desktop investigation did not include a review of the quality of care. The extent to which such a review is warranted, given the methodological issues with HSMR, remains an open question. Nevertheless SaTH did undertake an internal review of the quality of care by retrospective case-notes review and concluded that “To date, no significant concerns about quality of care have been identified” [31], furthermore “The Trust has also been rated as one of the best in the country for the patient’s view of the overall care they received.” and survival rates following bowel cancer surgery are amongst the highest in the country [32].
